# A Survey of Anxiety Sensitivity Index Levels in School-Aged Children With Asthma and a Study of Its Influencing Factors and Predictive Models

**DOI:** 10.62641/aep.2263

**Published:** 2026-10-15

**Authors:** Haiwei Zhao, Liman Hu, Bohao Li, Zishuo Liu, Qingyang Bao

**Affiliations:** ^1^Department of Pediatrics, Yueqing People's Hospital, 325600 Wenzhou, Zhejiang, China

**Keywords:** anxiety sensitivity index, asthma, children, influencing factor, predictive model

## Abstract

**Background:**

This study aimed to investigate the current status of anxiety sensitivity index levels in school-aged children with asthma, explore its influencing factors, establish a risk prediction model, and test its predictive effectiveness.

**Methods:**

We retrospectively analysed 400 school-aged children with asthma admitted to Yueqing People's Hospital between May 2020 and May 2025. The children were randomly divided into a construction group (n = 280) and a validation group (n = 120) at 7:3 ratio. Based on the anxiety sensitivity index levels, the construction group was further divided into a high-anxiety-sensitive group (n = 65) and a low-to-moderate-anxiety-sensitive group (n = 215); the validation group was similarly divided into a high-anxiety-sensitive group (n = 28) and a low-to-moderate-anxiety-sensitive group (n = 92). Clinical data were recorded. A nomogram prediction model was constructed using the R language software package. The model was evaluated using receiver operating characteristic (ROC) curves and calibration curves, and validated using the validation groups samples.

**Results:**

Multivariate analysis of the construction group showed that the main risk factors for high anxiety sensitivity index in school-aged children with asthma were: primary caregiver education level below high school, asthma control status of partial or uncontrolled, primary caregiver monthly income < CNY 4000, incomplete family situation, and Sleep Disorders Scale for Children (SDSC) score ≥39 (*p* < 0.05). The predictive model constructed from these factors showed good discrimination ability in internal validation, with an area under the curve (AUC) of 0.794 (95% confidence interval [CI]: 0.730–0.858) and a C-index of 0.818 (95% CI: 0.733–0.835). In the validation group, the AUC was 0.799 (95% CI: 0.706–0.892) and a C-index was 0.820 (95% CI: 0.717–0.831), indicating good external predictive power.

**Conclusions:**

The main risk factors for high anxiety sensitivity index in school-aged children with asthma are: primary caregiver education level below high school, asthma control status of partial or uncontrolled, primary caregiver monthly income < CNY 4000, incomplete family situation, and an SDSC score ≥39. The nomogram model constructed from these factors can effectively predict the risk of high anxiety sensitivity index in school-aged children with asthma.

## Introduction

Bronchial asthma is one of the most common chronic respiratory diseases in childhood, with a high prevalence worldwide, imposing a significant burden on children’s physical and mental health, learning and daily life, as well as families and society [1, 2]. School age is a critical stage for children’s physiological and psychological development and social functioning. During this period, asthma—a disease that recurs and often requires long-term management—not only causes physical discomfort such as coughing, wheezing and chest tightness, but may also cause a series of emotional and behavioural problems due to the unpredictability of attacks, restrictions on daily activities and the worry about emergencies [3, 4]. Anxiety sensitivity refers to an individual’s fearful belief about anxiety-related physical or psychological feelings and the tendency to catastrophically interpret these feelings as harmful and impending danger [5, 6]. It is not only a core maintaining factor for anxiety disorders but may also amplify the perception of physical symptoms in patients with chronic diseases, affecting disease management behaviour and quality of life [7, 8]. For school-aged children with asthma, symptoms such as shortness of breath and chest tightness overlap significantly with the physical manifestations of anxiety. High anxiety sensitivity may lead to hypervigilance and catastrophic interpretation of mild wheezing or discomfort, thereby exacerbating anxiety and even causing children to avoid normal school and social activities for fear of attacks, seriously affecting their physical and mental development [9, 10]. Although international research has begun to examine the role of anxiety sensitivity in chronic diseases, domestic studies in China have largely focused on general anxiety or depressive symptoms in children with asthma, with little systematic investigation into the factors influencing their levels of anxiety sensitivity. Therefore, this study aimed to investigate the current status of anxiety sensitivity indices in school-aged children with asthma, analyse the influencing factors, and develop a predictive model to provide a basis for the early identification of high-risk children.

## Materials and Methods

### Research Subjects

Between May 2020 and May 2025, Yueqing People’s Hospital enrolled 482 school-aged children with asthma in this study. Of these, 32 had other serious chronic physical or neurological conditions; 18 had experienced a major stressful event within the past month; 12 had guardians unable to cooperate with the survey; and 20 refused participation. Ultimately, 400 children were included in the retrospective analysis and randomly assigned to a construction group (n = 280) and a validation group (n = 120) at a 7:3 ratio. Data from the construction group were used to build the model, and data from the validation group were used to validate the model. Based on the anxiety sensitivity index, patients in the construction group were divided into a high-anxiety-sensitivity group (n = 65) and a low-to-moderate-anxiety-sensitivity group (n = 215). The validation group was similarly divided into a high-anxiety-sensitivity group (n = 28) and a low-to-moderate-anxiety-sensitivity group (n = 92). The inclusion criteria were as follows: (1) meeting the diagnostic criteria of the “Guidelines for the Diagnosis and Prevention of Bronchial Asthma in Children” [11]; (2) age 6–14 years; (3) disease duration ≥6 months; (4) informed consent from the child and primary guardian, with basic reading comprehension and communication skills; and (5) complete medical records available. The exclusion criteria were as follows: (1) comorbid serious chronic physical illnesses or clearly diagnosed mental and neurological diseases; (2) experiencing a major stress event within the past month; or (3) guardian has cognitive or mental disorders and is unable to cooperate with the investigation. This study was approved by the Ethics Committee of Yueqing People’s Hospital (Ethics approval number: YQYY202600009, Approval date: 29 January 2026). All procedures involving human participants were in accordance with the ethical standards of the institutional research committee and with the 1964 Helsinki declaration and its later amendments or comparable ethical standards.

### Assessment of Anxiety Sensitivity Index Level

Data on anxiety sensitivity index levels were extracted from the paediatric patients’ medical records. The Childhood Anxiety Sensitivity Index (CASI) scale, comprising 18 items each scored from 0 to 2 points (total range 0–36, with higher scores indicating higher anxiety sensitivity), was used for assessment [12]. Because no universally accepted clinical cutoff for the CASI exists in paediatric populations with asthma, we grouped children according to the distribution of scores in the construction cohort: the top 27% were designated as the high anxiety sensitivity group, the bottom 27% as the low group, and the remaining 46% as the moderate group. This 27/46/27 split follows a conventional method for creating contrasted groups based on score percentiles.

### Observation Indicators

Clinical data of the children were collected retrospectively from medical records, including gender, age, disease duration, education level of the primary caregiver, age of the primary caregiver, place of residence, payment method for medical expenses, family history of asthma, asthma control status, monthly income of the primary caregiver, family situation, sleep status of the children, and medication adherence of the child. Asthma control status was determined according to the following four items in the grading standard for asthma control level in children aged ≥6 years: daytime symptoms > 2 times/week; nighttime awakening due to asthma; use of emergency relief medication > 2 times/week; and activity limitation due to asthma. If three to four items are present, asthma is uncontrolled; if one to two items are present, asthma is partially controlled; and if none of the four symptoms are present, asthma is well controlled [13]. The children’s sleep status was assessed using the Sleep Disorders Scale for Children (SDSC), which contains 26 items covering six dimensions: sleep onset or maintenance disorder, sleep breathing disorder, sleep arousal disorder, sleep–wake transition disorder, excessive sleepiness disorder, and excessive sweating during sleep. Each item was scored on a Likert 5-point scale, with a total score range of 26 to 130. Higher scores indicate more serious the sleep disorder problem. A total score ≥39 indicates the presence of sleep disorder [14]. Medication adherence was assessed using the Medication Adherence Questionnaire-8 (MMAS-8), which has a maximum score of 8. Higher scores indicate better adherence. Medication adherence was divided into three levels: 8 points indicates excellent adherence, 6 to <8 points indicates good adherence, and <6 points indicates poor adherence [15].

### Statistical Analysis

Data analysis was performed using SPSS 21.0 software (IBM Corp., Armonk, NY, USA). The Kolmogorov–Smirnov test was used to test the normality of continuous variables. Normally distributed variables are expressed as mean ± standard deviation, and independent samples *t*-tests were used for comparisons between two groups. Non-normally distributed variables are expressed as median (interquartile range) [M (P25, P75)], and Mann–Whitney U test was used for between-group comparisons. Count data are expressed as percentages (%), and chi-square tests were used for between-group comparisons. Univariate analysis was performed on potential influencing factors of high anxiety sensitivity index levels in school-aged children with asthma. Variables with statistically significant differences were further included in binary logistic regression analysis, and receiver operating characteristic (ROC) curves were plotted to assess predictive power. The construction group was used to build the nomogram prediction model, and the validation group was used for external validation. All hypothesis tests were two-tailed, with *p* < 0.05 considered statistically significant.

## Results

### Results of the Anxiety Sensitivity Index Survey

Among the 400 children included in this retrospective study, 174 had low CASI scores, 133 had moderate scores, and 93 had high scores. In the construction group, 122 children had low scores, 93 moderate scores, and 65 high scores; in the validation group, 52 had low scores, 40 moderate scores, and 28 high scores.

### General Information

The 400 patients were randomly divided into a construction group (n = 280) and a validation group (n = 120) at a 7:3 ratio. Comparison of general characteristics between the two groups showed no statistically significant differences (*p* > 0.05), indicating comparability (Table 1).

**Table 1.** **Comparison of clinical data between the two groups [$\bar{x}$ ± s / n (%)]**.

Variable		Construction group (n = 280)	Validation group (n = 120)	χ^2^ / *t*	*p*
Gender				0.125	0.724
	Male	164 (58.57)	68 (56.67)		
	Female	116 (41.43)	52 (43.33)		
Age		8.30 ± 2.19	8.36 ± 2.11	0.300	0.764
Course of disease		1.57 ± 0.54	1.52 ± 0.56	0.836	0.404
Primary caregiver’s educational level				0.138	0.710
	Below high school	72 (25.71)	33 (27.50)		
	High school and above	208 (74.29)	87 (72.50)		
Primary caregiver age		35.76 ± 5.58	35.83 ± 5.47	0.087	0.931
Place of residence				0.039	0.844
	Rural areas	137 (48.93)	60 (50.00)		
	City	143 (51.07)	60 (50.00)		
Medical expense payment methods				0.302	0.860
	Publicly funded medical care	51 (18.21)	24 (20.00)		
	Insurance Medical	30 (10.71)	14 (11.67)		
	Out-of-pocket medical care	199 (71.07)	82 (68.33)		
Family history of asthma				0.168	0.682
	Have	31 (11.07)	15 (12.50)		
	None	249 (88.93)	105 (87.50)		
Asthma control				0.278	0.598
	Partial control or no control	185 (66.07)	76 (63.33)		
	Well controlled	95 (33.93)	44 (36.67)		
Primary caregiver’s monthly income (CNY)				0.135	0.713
	<4000	95 (33.93)	43 (35.83)		
	≥4000	185 (66.07)	77 (64.17)		
Family situation				0.313	0.576
	Whole	174 (62.14)	71 (59.17)		
	Incomplete	106 (37.86)	49 (40.83)		
SDSC score (points)				0.040	0.842
	<39	165 (58.93)	72 (60.00)		
	≥39	115 (41.07)	48 (40.00)		
MMAS-8 rating (points)				0.105	0.949
	8	86 (30.71)	40 (33.33)		
	6–<8	96 (34.29)	38 (31.67)		
	<6	98 (35.00)	42 (35.00)		

Note: SDSC, Sleep Disorders Scale for Children; MMAS-8, Medication Adherence Questionnaire-8. 1 USD = 7.1950 CNY.

### Univariate Analysis of the High Anxiety Sensitivity Index Levels in School-Aged Asthmatic Children in the Construction Group

No statistically significant differences were observed between the two groups in gender, age, age of primary caregiver, place of residence, payment method for medical expense, family history of asthma, or MMAS-8 score (*p* > 0.05). However, significant differences were found in five indicators: primary caregiver education level, asthma control status, primary caregiver monthly income, family situation, and SDSC score (*p* < 0.05, Table 2).

**Table 2.** **Univariate analysis of the high anxiety sensitivity index levels in school-aged asthmatic children in the construction group [$\bar{x}$ ± s / n (%)]**.

Variable		High anxiety and sensitivity group (n = 65)	Low and moderate anxiety sensitivity groups (n = 215)	χ^2^ / *t*	*p*
Gender				0.095	0.758
	Male	37 (56.92)	127 (59.07)		
	Female	28 (43.08)	88 (40.93)		
Age		8.36 ± 2.18	8.29 ± 2.22	0.339	0.735
Disease duration (years)		1.52 ± 0.51	1.58 ± 0.55	0.778	0.437
Primary caregiver’s educational level				15.832	<0.001
	Below high school	29 (44.62)	43 (20.00)		
	High school and above	36 (55.38)	172 (80.00)		
Primary caregiver age		35.84 ± 5.61	35.77 ± 5.59	0.033	0.973
Place of residence				0.387	0.534
	Rural areas	34 (52.31)	103 (47.91)		
	City	31 (47.69)	112 (52.09)		
Medical expense payment methods				1.796	0.407
	Publicly funded medical care	15 (23.08)	36 (16.74)		
	Insurance Medical	8 (12.31)	22 (10.23)		
	Out-of-pocket medical care	42 (64.61)	157 (73.03)		
Family history of asthma				0.665	0.416
	Have	9 (13.85)	22 (10.23)		
	None	56 (86.15)	193 (89.77)		
Asthma control				14.981	<0.001
	Partial control or no control	30 (46.15)	155 (72.09)		
	Well controlled	35 (53.85)	60 (27.91)		
Primary caregiver’s monthly income (CNY)				10.710	0.001
	<4000	33 (50.77)	62 (28.84)		
	≥4000	32 (49.23)	153 (71.16)		
Family situation				17.643	<0.001
	Whole	26 (40.00)	148 (68.84)		
	Incomplete	39 (60.00)	67 (31.16)		
SDSC score (points)				14.651	<0.001
	<39	25 (38.46)	140 (65.12)		
	≥39	40 (61.54)	75 (34.88)		
MMAS-8 rating (points)				0.447	0.800
	8	19 (29.23)	67 (31.17)		
	6∼<8	21 (32.31)	75 (34.88)		
	<6	25 (38.46)	73 (33.95)		

Note: SDSC, Sleep Disorders Scale for Children; MMAS-8, Medication Adherence Questionnaire-8. 1 USD = 7.1950 CNY.

### Multivariate Analysis of High Anxiety Sensitivity Index Levels in School-Aged Asthmatic Children in the Construction Group

Binary logistic regression analysis showed that the main risk factors for high anxiety sensitivity index in school-aged children with asthma were: primary caregiver education level below high school, asthma control status of partial or uncontrolled, primary caregiver monthly income < CNY 4000, incomplete family situation, and SDSC score ≥39 (Tables 3 and 4).

**Table 3.** **Explanation and values of independent variables in logistic regression analysis**.

Variable	Variable description	Assignment status
Primary caregiver’s educational level	Binary variable	Below high school = 1, High school and above = 2
Asthma control	Binary variable	Partial control or no control = 1, Well controlled = 2
Primary caregiver’s monthly income (CNY)	Binary variable	<4000 CNY = 1, ≥4000 CNY = 2
Family situation	Binary variable	Incomplete = 1, Complete = 2
SDSC score (points)	Binary variable	≥39 points=1, <39 points=2

Note: SDSC, Sleep Disorders Scale for Children. 1 USD = 7.1950 CNY.

**Table 4.** **Multivariate analysis of the high anxiety sensitivity index levels in school-aged asthmatic children in the construction group**.

Variable	Regression coefficient	Standard error	Wald	*p*-value	OR value	95% CI
Primary caregiver’s educational level	1.357	0.352	14.858	<0.001	3.885	1.949–7.747
Asthma control	1.046	0.332	9.897	0.002	2.845	1.483–5.457
Primary caregiver’s monthly income (CNY)	1.127	0.340	10.967	0.001	3.085	1.584–6.010
Family situation	1.061	0.333	10.119	0.001	2.889	1.503–5.554
SDSC score (points)	0.841	0.328	6.570	0.010	2.318	1.219–4.409

Note: SDSC, Sleep Disorders Scale for Children; CI, confidence interval; OR, odds ratio. 1 USD = 7.1950 CNY.

### Construction of the Column Chart Prediction Model

Based on multivariate analysis results, a predictive model was constructed that included primary caregiver education level, asthma control status, primary caregiver monthly income, family situation and SDSC score. A nomogram was then plotted, with the length of the line segment for each predictor reflecting its contribution to the outcome, and the upper scale representing the individual item score. The total score was obtained by summing the scores corresponding to the values of each variable. The total score corresponds to the predicted probability of a high anxiety sensitivity index level in school-aged children with asthma (Fig. 1).

**Fig. 1.**
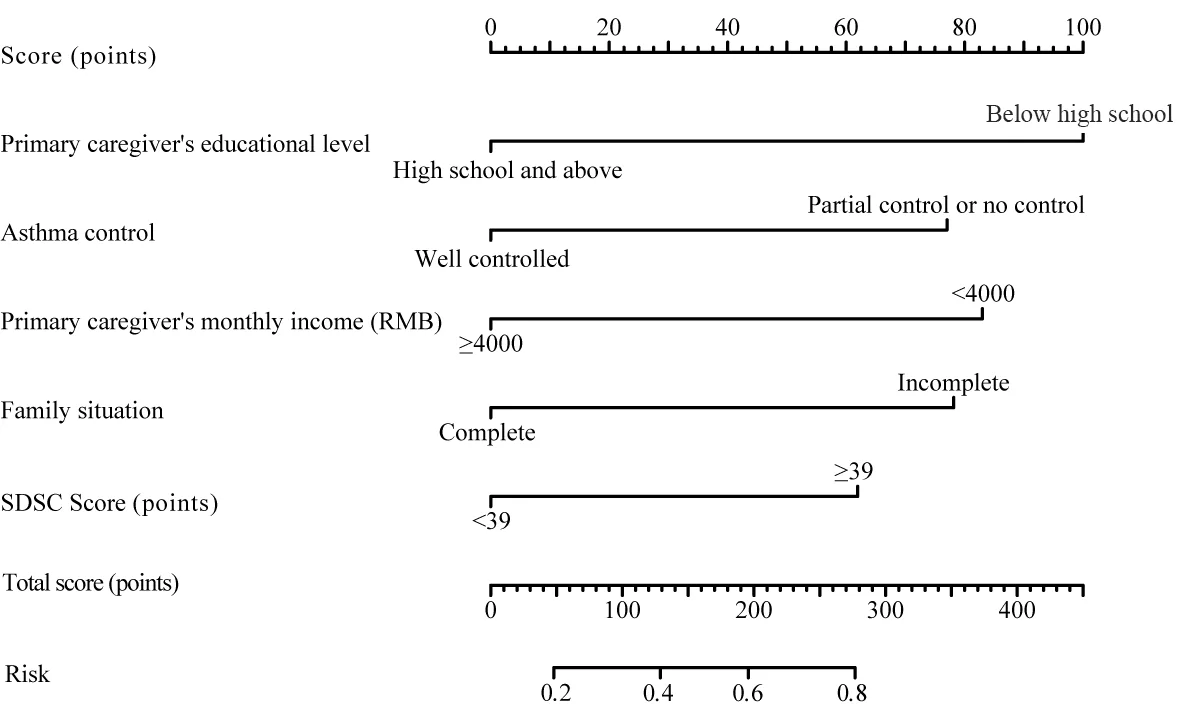
**Risk nomogram of high anxiety sensitivity index in school-aged asthmatic children**. SDSC, Sleep Disorders Scale for Children.

### ROC Curve Analysis of Relevant Variables in Construct and Validation Groups

Primary caregiver education level, asthma control status, monthly income, family situation, and SDSC score were the influencing factors for high anxiety sensitivity index level in school-aged children with asthma, with area under the curves (AUCs) of 0.623, 0.630, 0.610, 0.644 and 0.633, respectively (*p* < 0.05). The ROC curve analysis results for the construction group showed an AUC of 0.794, with a 95% confidence interval (CI) of 0.730–0.858. When the predicative model constructed from the construction group was applied to the validation group samples for external validation, the AUC was 0.799 (95% CI: 0.706–0.892, Table 5 and Fig. 2).

**Table 5.** **ROC curve analysis of relevant variables in construct and validation groups**.

variable	AUC	Optimal cutoff value	Youden index	SE	95% CI	*p*-value	Sensitivity (%)	Specificity (%)
Primary caregiver’s educational level	0.623*	1	0.246	0.038	0.557–0.690	0.002	80.00	44.61
Asthma control	0.630*	1	0.259	0.042	0.562–0.698	0.001	72.09	53.84
Primary caregiver’s monthly income (CNY)	0.610*	1	0.219	0.035	0.541–0.678	<0.001	71.16	50.76
Family situation	0.644*	1	0.288	0.045	0.577–0.712	0.001	68.83	60.00
SDSC score (points)	0.633*	1	0.266	0.033	0.566–0.701	<0.001	61.54	65.12
Construction group	0.794	0.714	0.549	0.036	0.730–0.858	<0.001	71.70	80.32
Validation group	0.799	0.801	0.544	0.030	0.706–0.892	<0.001	90.00	64.44

Note: Compared with the AUC of Construction group, *p < 0.05; SDSC, Sleep Disorders Scale for Children; SE, standard error; CI, confidence interval; AUC, area under the curve; ROC, receiver operating characteristic. 1 USD = 7.1950 CNY.

**Fig. 2.**
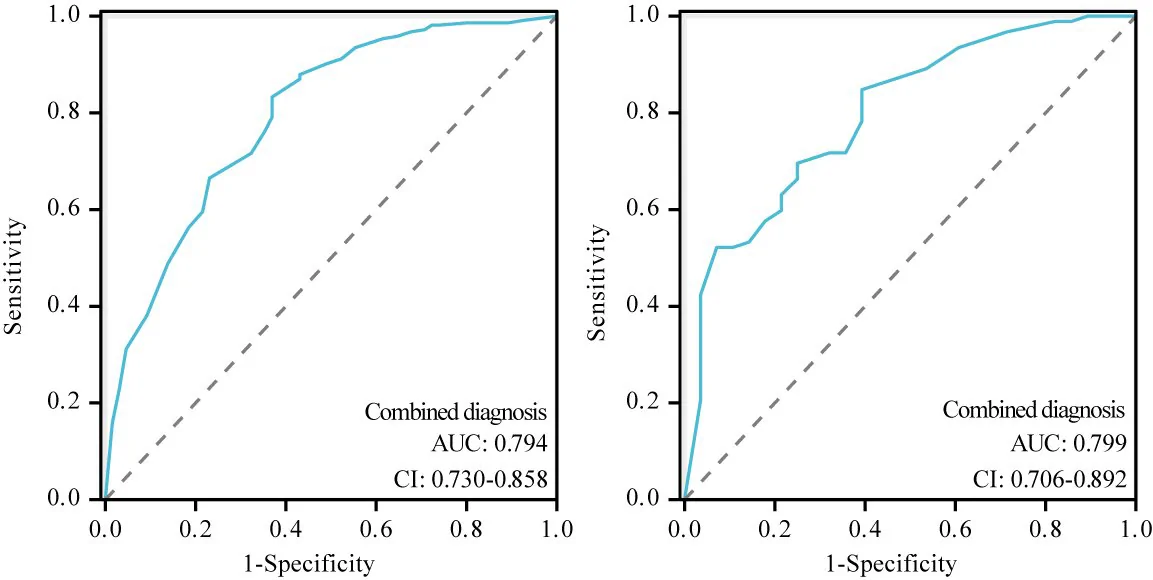
**ROC curves of the construction group (left) and the validation group (right)**. Note: ROC curves of the joint predictive model (including primary caregiver’s education level, asthma control status, primary caregiver’s monthly income, family situation, and SDSC score) predicting the risk of high anxiety sensitivity in both the construct and validation groups. ROC, receiver operating characteristic; SDSC, Sleep Disorders Scale for Children; CI, confidence interval; AUC, area under the curve.

### Evaluation of Nomogram Model

The calibration curve for the construction group had a C-index of 0.818 (95% CI: 0.733–0.835), and the calibration curve closely matched the ideal curve. The calibration curve for the validation group had a C-index of 0.820 (95% CI: 0.717–0.831), indicating good calibration (Fig. 3).

**Fig. 3.**
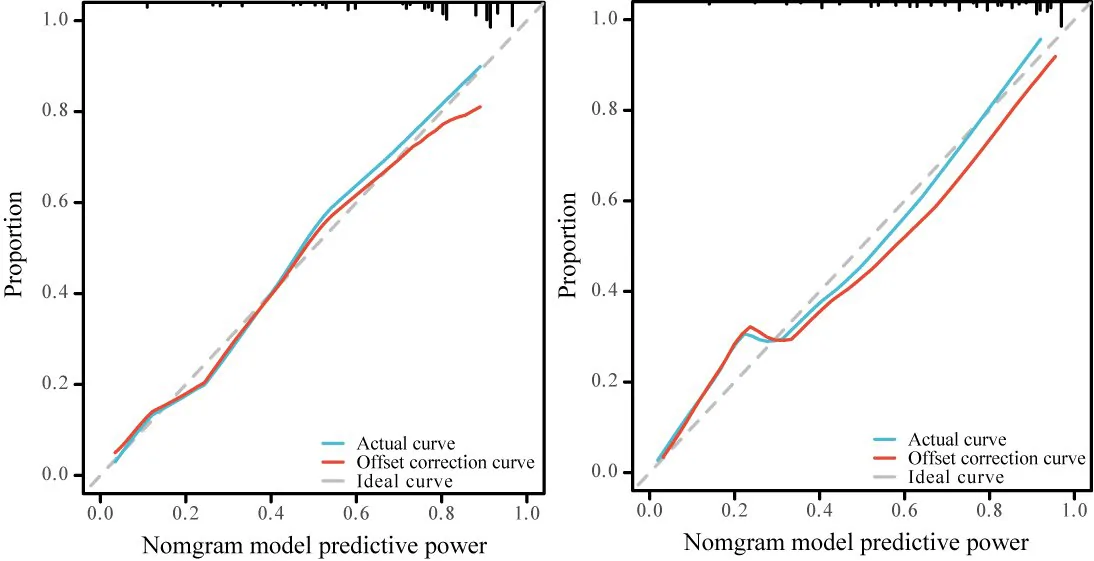
**Calibration curves for the construction group (left) and the calibration curves for the validation group (right)**.

## Discussion

As the most common chronic respiratory disease in childhood, bronchial asthma has gradually evolved from simple symptom control to comprehensive health management covering physiological, psychological and social functions. This study found significant differences in anxiety sensitivity levels among school-aged children with asthma, with some children having a high anxiety sensitivity index. Anxiety sensitivity refers to the negative effect that patients believe anxiety-related feelings have on their physical and mental health and society functioning, causing them to feel uneasy and panicked about anxiety symptoms. It can reflect the patient’s psychological state. A higher anxiety sensitivity index is associated with poorer health outcomes in children with asthma. The results of this study show that primary caregiver education level below high school, asthma control status of partially controlled or uncontrolled, primary caregiver monthly income < CNY 4000, incomplete family situation, and SDSC score ≥39 are the main risk factors for high anxiety sensitivity index in school-aged children with asthma. The reasons are analysed as follows: (1) Caregivers with lower education levels may have more difficulty in understanding asthma, adhering to standardized treatment plans and comprehending health information. They may rely more on experience or information from informal channels, feeling helpless and anxious about the unpredictability and long-term nature of the disease [16]. This anxiety and vague understanding of disease management are transmitted to children through daily interactions, amplifying their fear of physical symptoms [17]. At the same time, these caregivers may be less likely to use scientific and rational methods to explain the disease to their children, thereby indirectly increasing the children’s anxiety sensitivity [18]. (2) Poor asthma control is the core disease-related factor leading to high anxiety sensitivity. Repeated or persistent coughing, wheezing and other symptoms constantly reinforce the child’s negative experience and catastrophic association with the feeling of difficulty breathing [19]. Poor control means increased unpredictability of symptom attacks, leaving the child in a state of vigilance and worry about “when will it recur”. This continuous psychological stress significantly increases their sensitivity and fear of related physical signals [20, 21]. (3) Long-term management of asthma requires stable economic income. Economic pressure may make families more cautious or even financially strained in their treatment choices, worrying about medical expenses, which increases the psychological burden on the primary caregiver [22, 23]. The pervasive economic pressure and uncertainty about the future can create a tense and uneasy family emotional atmosphere [24]. Children can be keenly perceive this pressure and internalize it into their own insecurity, which manifests as a higher level of anxiety sensitivity. (4) An intact family can help children obtain sufficient psychological comfort and family care, which can help reduce psychological stress and gradually foster correct and positive cognition and behaviour [25, 26]. Single-parent or divorced families may make it difficult for children to receive timely and sufficient emotional responses and comfort when they feel physical discomfort or fear, leading them to be more inclined to face and catastrophize these feelings alone, thereby exacerbating their level of anxiety sensitivity [27, 28]. (5) A vicious cycle may exist between sleep disturbances and anxiety sensitivity. From a neurological perspective, sleep deprivation can weaken the functional connectivity between the prefrontal cortex and the amygdala, impairing the prefrontal cortex’s inhibitory control over the amygdala and thus increasing susceptibility to emotional responses [29]. Furthermore, sleep disturbances may also alter the neural substrates underlying mood regulation through pro-inflammatory cytokine secretion. Study by Uy *et al*. [30] have found that sleep deprivation and elevated inflammation levels can synergistically affect the prefrontal-limbic circuit, thereby exacerbating anxiety symptoms. For children with asthma, the potential for nocturnal hypoxia interacts with sleep disturbances, further aggravating these neurophysiological changes and increasing anxiety sensitivity.

Using univariate and multivariate regression analyses, this study systematically identified independent factors associated with high anxiety sensitivity scores in school-aged children with asthma and used these findings to develop a risk-prediction nomogram model. This model quantifies the contribution of each influencing factor via an intuitive scoring system to enable individualized risk assessment. The model exhibits good discrimination, with a C-index of 0.818 (range 0.733–0.835). Its application can provide a more accurate risk assessment of high anxiety sensitivity index levels in school-aged children with asthma, thereby enabling targeted preventative measures. This study has several limitations. Firstly, the classification of low, moderate, and high anxiety sensitivity relied on the 27th and 73rd percentiles of CASI scores in the constructed cohort. This data-based grouping method is sample-dependent and may not be directly comparable to studies using predefined clinical cutoff values or continuous CASI score processing. Future research should determine effective CASI cutoff values applicable to this population. Secondly, the retrospective, single-centre study design and the exclusion of children with incomplete medical records may have introduced selection bias. Therefore, these findings need further validation in prospective, multicentre studies.

## Conclusions

This study shows that the main risk factors for a high anxiety sensitivity index in school-aged children with asthma are: primary caregiver education level below high school, asthma control status of partially or uncontrolled, primary caregiver monthly income < CNY 4000, incomplete family situation, and SDSC score ≥39. The nomogram model established from these factors demonstrates good predictive power.

## Availability of Data and Materials

All experimental data included in this study can be obtained by contacting the corresponding author if needed.
